# Activity landscape image analysis using convolutional neural networks

**DOI:** 10.1186/s13321-020-00436-5

**Published:** 2020-05-18

**Authors:** Javed Iqbal, Martin Vogt, Jürgen Bajorath

**Affiliations:** Department of Life Science Informatics, B-IT, LIMES Program Unit Chemical Biology and Medicinal Chemistry, Rheinische Friedrich-Wilhelms-Universität, Endenicher Allee 19c, 53115 Bonn, Germany

**Keywords:** Activity landscape, Structure–activity relationships, Image processing, Image classification, Machine learning, Convolutional neural network, Landscape topology, Feature extraction

## Abstract

Activity landscapes (ALs) are graphical representations that combine compound similarity and activity data. ALs are constructed for visualizing local and global structure–activity relationships (SARs) contained in compound data sets. Three-dimensional (3D) ALs are reminiscent of geographical maps where differences in landscape topology mirror different SAR characteristics. 3D AL models can be stored as differently formatted images and are thus amenable to image analysis approaches, which have thus far not been considered in the context of graphical SAR analysis. In this proof-of-concept study, 3D ALs were constructed for a variety of compound activity classes and 3D AL image variants of varying topology and information content were generated and classified. To these ends, convolutional neural networks (CNNs) were initially applied to images of original 3D AL models with color-coding reflecting compound potency information that were taken from different viewpoints. Images of 3D AL models were transformed into variants from which one-dimensional features were extracted. Other machine learning approaches including support vector machine (SVM) and random forest (RF) algorithms were applied to derive models on the basis of such features. In addition, SVM and RF models were trained using other features obtained from images through edge filtering. Machine learning was able to accurately distinguish between 3D AL image variants with different topology and information content. Overall, CNNs which directly learned feature representations from 3D AL images achieved highest classification accuracy. Predictive performance for CNN, SVM, and RF models was highest for image variants emphasizing topological elevation. In addition, SVM models trained on rudimentary images from edge filtering classified such images with high accuracy, which further supported the critical role of altitude-dependent topological features for image analysis and predictions. Taken together, the findings of our proof-of-concept investigation indicate that image analysis has considerable potential for graphical SAR exploration to systematically infer different SAR characteristics from topological features of 3D ALs.
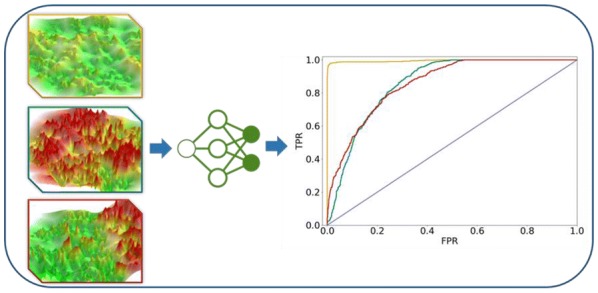

## Introduction

Activity landscapes (ALs) are defined as graphical representations that integrate compound similarity and activity relationships [1, 2]. ALs graphically represent active compounds in biologically relevant chemical space, making it possible to visualize structure–activity relationships (SARs) and identify key compounds and SAR determinants [1–8]. A variety of AL representations of different design and complexity have been introduced to visualize SARs. These include structure–activity similarity maps, other two-dimensional (2D) ALs, three-dimensional (3D) AL models, and molecular network representations [1–8]. 3D ALs can be rationalized to result from a two-dimensional (2D) projection of chemical feature space, producing a plane where compounds are separated by varying distances, to which compound potency is added as a third dimension. From sparsely distributed potency measurements, an activity hyper-surface is interpolated [3, 8]. Compounds that are similar to each other and neighbors in chemical space but have large differences in potency form activity cliffs (ACs) [1–3], which are prominent features of 3D AL models. Such 3D ALs are reminiscent of geographical maps with varying landscape topologies [3, 8]. In 3D ALs, the activity hyper-surface can be color-coded by compound potency using a color gradient, which further emphasizes different topologies. In gently sloped or smooth regions, gradual changes in chemical structure are accompanied by moderated changes in potency, which corresponds to SAR continuity [1–3]. By contrast, in rugged regions, small chemical changes lead to significant potency variations, corresponding to SAR discontinuity [1–3]. Here, ACs represent the apex of SAR discontinuity. By design, 3D ALs are descriptive in nature and are typically qualitatively analyzed. Only very few studies have thus far attempted to use AL models for compound potency predictions [4, 8].

3D AL models can be visualized and analyzed from different viewpoints and perspectives. Hence, visualization yields images with different characteristics that can be subjected to image processing methods. Thus far, however, AL visualizations have not been analyzed and compared using such approaches. Therefore, we have asked the question if 3D ALs with different topological features representing different SAR characteristics could be distinguished from each other and classified through image processing; the major topic of this study.

In recent years, deep learning has made a large impact on image processing. In particular, convolutional neural networks (CNNs) have become one of the preferred machine learning approaches for image analysis due to their ability to extract patterns from low-resolution data representations in so-called convolutional layers [9, 10]. CNNs are deep neural networks with one or more convolutional layers encoding locality information in the network structure [9, 10]. The design of CNNs renders them well-suited for processing of spatial and temporal data such as images, audio, or video signals. CNNs achieved higher performance level than other computational approaches in recognizing histopathological [11], magnetic resonance [12], medical X-ray [13], computer tomography, [14] and fundus images [15]. CNNs are also gaining increasing attention in chemical informatics and drug discovery, albeit in different contexts. For example, CNNs and random forest (RF) [16] models have been applied to predict cancer cell line sensitivity and compound potency [17] or compound toxicity [18] using 2D structural representations in image formats. CNNs have also been employed to model bioactivity profiles using 2D images [19, 20]. Other studies investigated molecular mechanism of action based on bioactivity profiles using images from high-content screening [21–23].

In addition to CNNs, various studies have shown that other machine learning approaches like support vector machine (SVM) [24] modeling can also classify images using raw pixel intensities or extracted image features [25–30]. In addition, RF can also accurately classify high-dimensional image data [31, 32].

However, the application of CNNs or other machine learning-based image processing methods to ALs for SAR visualization and analysis has thus far not been investigated. For machine learning methods, suitable representations are required to represent data sets of varying size in a unified and standardized format to enable direct comparison. Images generated from 3D ALs are well suited because they retain the pairwise similarity relationships between compounds and account for potency values as topographical features and/or using color gradients. Images can be generated from different viewpoints ranging from top-down views of ALs to elevated or profile views where SARs become visible as peaks and valleys. Top-down views essentially yield heatmap representations if color gradients are used, as further discussed below.

In our current study, 3D AL images have been analyzed and classified using CNNs, RF, and SVM. Different projection methods and image encodings of varying resolution and information content have been generated to capture 3D AL topology in different ways and determine which factors are responsible for accurate image classification. Therefore, image variants with successively reduced information content have also been generated and investigated.

CNN, RF, and SVM models were found to be capable of correctly classifying AL image variants with different topology on the basis of structure and pixel intensity information. CNNs learning feature representations yielded overall most accurate predictions. However, RF and SVM models trained on pre-defined lower-level feature representations were also predictive. The analysis identified topological features that were of critical relevance for image classification. Taken together, our findings revealed that images of 3D ALs for SAR visualization can be distinguished through machine learning on the basis of characteristic topological features, which provides a new methodological framework for direct comparison of AL models of compound data sets of different composition and comparative SAR analysis of large data sets.

## Concepts and methods

### Compound activity classes

For 3D AL image analysis, 38 compound activity classes were selected from ChEMBL version 23 [33]. For each class, more than 500 compounds with pK_i_ potency measurements were available. Intra-class potency variations spanned several orders of magnitude. In addition, the potency value distribution of each class had an interquartile range covering at least one order of magnitude [34]. Table 1 summarizes the composition of each activity class and provides potency range statistics. Reported are final compound numbers after similarity filtering, as further described below.

**Table 1:** Activity classes

ChEMBL target ID	Target name	No. compounds	Potency [pK_i_]		IQR (Q1–Q3)
			Min	Max	
CHEMBL204	Coagulation factor II	1099	1.00	12.19	5.30–7.47
CHEMBL205	Carbonic anhydrase 2	2701	0.60	11.10	6.24–8.04
CHEMBL214	Serotonin receptor 1A	1936	0.36	10.85	6.74–8.29
CHEMBL217	Dopamine D2 receptor	3427	2.85	10.57	6.29–7.49
CHEMBL218	Cannabinoid receptor 1	1938	3.79	10.10	5.95–7.62
CHEMBL219	Dopamine D4 receptor	1086	4.74	10.52	6.43–7.87
CHEMBL222	Sodium-dependent noradrenaline transporter	1000	2.26	9.52	5.86–7.54
CHEMBL224	Serotonin receptor 2A	1967	3.51	11.00	6.56–8.12
CHEMBL225	Serotonin receptor 2C	1085	3.51	9.70	6.23–7.70
CHEMBL226	Adenosine receptor A1	2829	4.12	12.23	5.87–7.14
CHEMBL229	Alpha-1A adrenergic receptor	594	4.04	10.44	6.90–8.40
CHEMBL233	Mu-type opioid receptor	2009	4.20	11.80	6.37–8.33
CHEMBL234	Dopamine D3 receptor	2518	4.17	10.00	6.79–8.40
CHEMBL236	Delta-type opioid receptor	1604	3.72	10.68	6.00–8.08
CHEMBL237	Kappa-type opioid receptor	1853	4.09	11.52	6.45–8.49
CHEMBL238	Sodium-dependent dopamine transporter	850	2.14	9.40	5.60–7.37
CHEMBL240	Potassium voltage-gated channel subfamily H_2	1053	3.89	9.55	5.29–6.44
CHEMBL245	Muscarinic acetylcholine receptor M3	609	4.11	10.30	6.70–9.10
CHEMBL251	Adenosine receptor A2a	3305	3.92	11.38	6.05–7.67
CHEMBL253	Cannabinoid receptor 2	2605	0.63	10.72	6.24–7.99
CHEMBL255	Adenosine receptor A2b	1265	3.37	9.80	6.30–7.82
CHEMBL256	Adenosine receptor A3	2567	1.32	11.00	6.16–7.84
CHEMBL261	Carbonic anhydrase 1	2657	0.56	11.00	5.34–7.09
CHEMBL264	Histamine H3 receptor	2323	4.07	10.60	7.21–8.70
CHEMBL344	Melanin-concentrating hormone receptor 1	1187	3.57	9.77	6.90–8.01
CHEMBL1800	Corticotropin-releasing factor receptor 1	673	4.26	9.66	6.58–8.14
CHEMBL1833	5-hydroxytryptamine receptor 2B	695	5.00	9.96	6.13–7.40
CHEMBL2014	Nociceptin receptor	839	4.40	10.70	7.09–8.52
CHEMBL3155	5-hydroxytryptamine receptor 7	1111	3.30	10.00	6.53–7.95
CHEMBL3242	Carbonic anhydrase 12	2008	3.08	9.62	6.92–8.23
CHEMBL3371	5-hydroxytryptamine receptor 6	2134	1.38	10.40	7.03–8.52
CHEMBL3594	Carbonic anhydrase 9	2347	1.34	9.92	6.61–8.04
CHEMBL3759	Histamine H4 receptor	887	2.85	10.40	5.98–7.59
CHEMBL4005	Serine/threonine protein kinase PIK3CA	882	4.65	10.52	7.01–8.46
CHEMBL4550	Arachidonate 5-lipoxygenase-activating protein	1318	5.60	9.40	6.75–8.21
CHEMBL4792	Orexin receptor type 2	1444	4.96	10.15	6.13–7.57
CHEMBL5071	Prostaglandin D2 receptor 2	794	4.48	10.00	6.49–8.41
CHEMBL5113	Orexin receptor type 1	1249	4.19	9.80	5.47–7.19

The table summarizes the composition of 38 activity classes used for 3D AL modeling. IQR represents the interquartile range of the potency value distribution of each data set

### Molecular representation and similarity assessment

For similarity assessment, the extended-connectivity fingerprint with bond diameter 4 (ECFP4) [35] was calculated for each compound. ECFP4 is a topological feature set fingerprint comprising layered atom environments and represents a gold standard in the field. ECFP4 feature sets were folded into a fixed-length 1024-bit representation [35]. As a similarity metric, the Tanimoto coefficient (Tc) was used to quantify pairwise compound similarity [36]. The Tc is defined as:

$${\text{Tc}}\left( {A,B} \right) = \frac{{\left| {A \cap B} \right|}}{{\left| A \right| + \left| B \right| - \left| {A \cap B} \right|}}$$

where $A$, $B$ are fingerprints of compounds A and B, respectively. Corresponding Tanimoto distance was obtained by calculating the complement $1 - {\text{Tc}}\left( {A,B} \right)$.

Initially assembled activity classes were subjected to similarity filtering and only compounds were retained that possessed an ECFP4 Tc similarity of at least 0.4 to at least one other compound from the same activity class. Filtering was applied to eliminate singletons from the data sets that had no or only very weak structural relationships with other compounds (and hence did not contribute to SARs). Fingerprint and similarity calculations were performed using in-house Python scripts and the OpenEye chemistry toolkit [37].

### 3D activity landscapes

For generating 3D AL models, ECFP4 space was projected on a 2D plane, compound potency values were added as the third dimension and from these values, a coherent potency hyper-surface was interpolated. Different projection methods for 3D AL design have previously been investigated [8] and two methods, multi-dimensional scaling (MDS) [38] and Neuroscale [39], were found to be preferred for retaining original similarity relationships for SAR visualization. Therefore, these approaches were used herein. For projection, both MDS and Neuroscale apply stress functions based on pairwise Tanimoto distances between compounds. Neuroscale projects compounds using a radial basis function (RBF) neural network. For each Neuroscale model, the number of RBFs was optimized using sevenfold cross validation.

Hyper-surface interpolation was carried out using Gaussian process regression (GPR) [40, 41]. The resulting surface was colored according to the compound potency using a color gradient from green over yellow to red. For all images, the same color gradient was applied according to which a pK_i_ value of 5.75 (and below) corresponded to green, the pK_i_ range 5.76–8.74 pK_i_ to yellow, and a pK_i_ of 8.75 (or above) to red.

### Reference landscapes

Smooth and rugged regions represent major topological features of 3D ALs that correspond to different SAR phenotypes [3]. In smooth regions, gradual changes in molecular structure are accompanied by moderate changes in potency, which represents SAR continuity. By contrast, in rugged regions, small structural changes lead to large potency variations. This corresponds to SAR discontinuity and leads to the formation of ACs. In many activity classes, continuous and discontinuous SAR components co-exist and are combined in different ways, giving rise to globally heterogeneous SARs [42, 43]. Such SAR heterogeneity is quantitatively accounted for using numerical SAR analysis functions such as the SAR Index [42]. In 3D AL models, SAR heterogeneity is represented by co-occurrence of smooth and rugged regions in different topological constellations.

To establish proof-of-concept for image classification, two reference AL models were generated for the 3D AL of each activity class in which SAR continuity/smoothness and discontinuity/ruggedness were increased, respectively, relative to the original 3D AL. Accordingly, these 3D AL variants were termed smooth and rugged reference (Ref-)ALs, respectively.

Smooth Ref-ALs were generated by selecting compounds that fell into the 2nd and 3rd quartile, i.e. the interquartile range, of the potency distribution of each activity class. Rugged Ref-ALs were obtained by considering septiles of the potency distribution and selecting compounds falling into the 1st, 3rd, 5th, and 7th septile. The resulting Ref-ALs contained about half and 4/7th the original number of compounds per class, respectively, which consistently amounted to more than 250 compounds per Ref-AL. Rugged Ref-ALs retained the potency range of the original ALs, whereas the potency of smooth Ref-ALs was reduced to the interquartile range, as reported in Table 1. It varied from ten- to 100-fold differences for most data sets while five sets had a larger than 100-fold interquartile range. As further discussed below, original 3D ALs of all 38 activity classes were generally heterogeneous in nature and were designated accordingly. Hence, for the generation of classification models, smooth and rugged Ref-ALs were distinguished from heterogeneous 3D ALs of original compound data sets, hence yielding three categories of 3D AL models for image generation.

### Activity landscape images

**Fig. 1:**
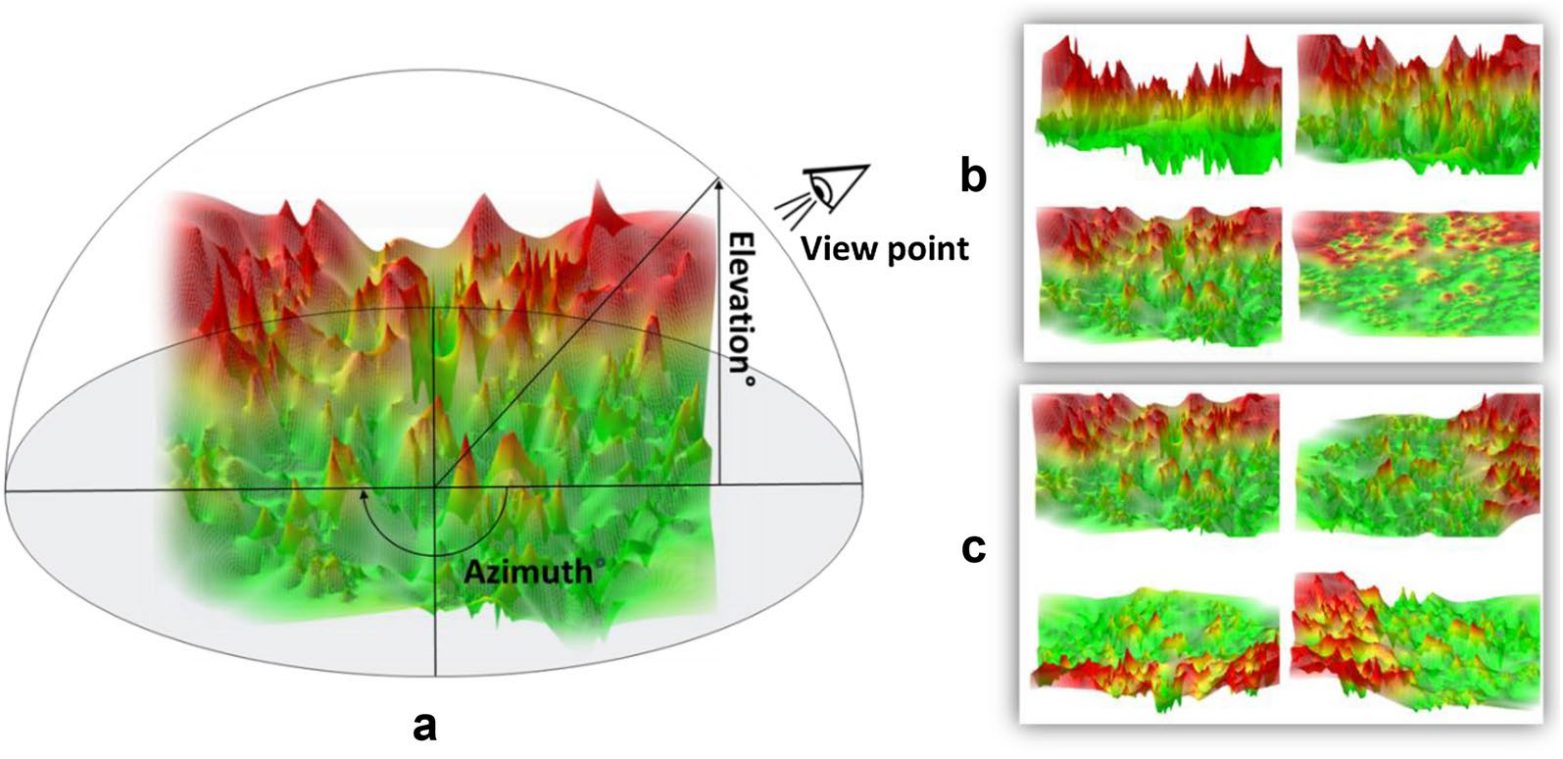
Different activity landscape views. For all activity classes, multiple 3D AL images were generated with varying azimuth and elevations settings. As an example, images with different views of a 3D AL are shown for activity class ChEMBL204 on the basis of Neuroscale projection. **a** illustrates that modification of the azimuth and elevation angle provide different views of a 3D AL. **b** shows 3D AL images with elevation angles of 0°, 35°, 65° and 90° and **c** images with azimuth angles of 0°, 90°, 180° and 270°

**Fig. 2:**
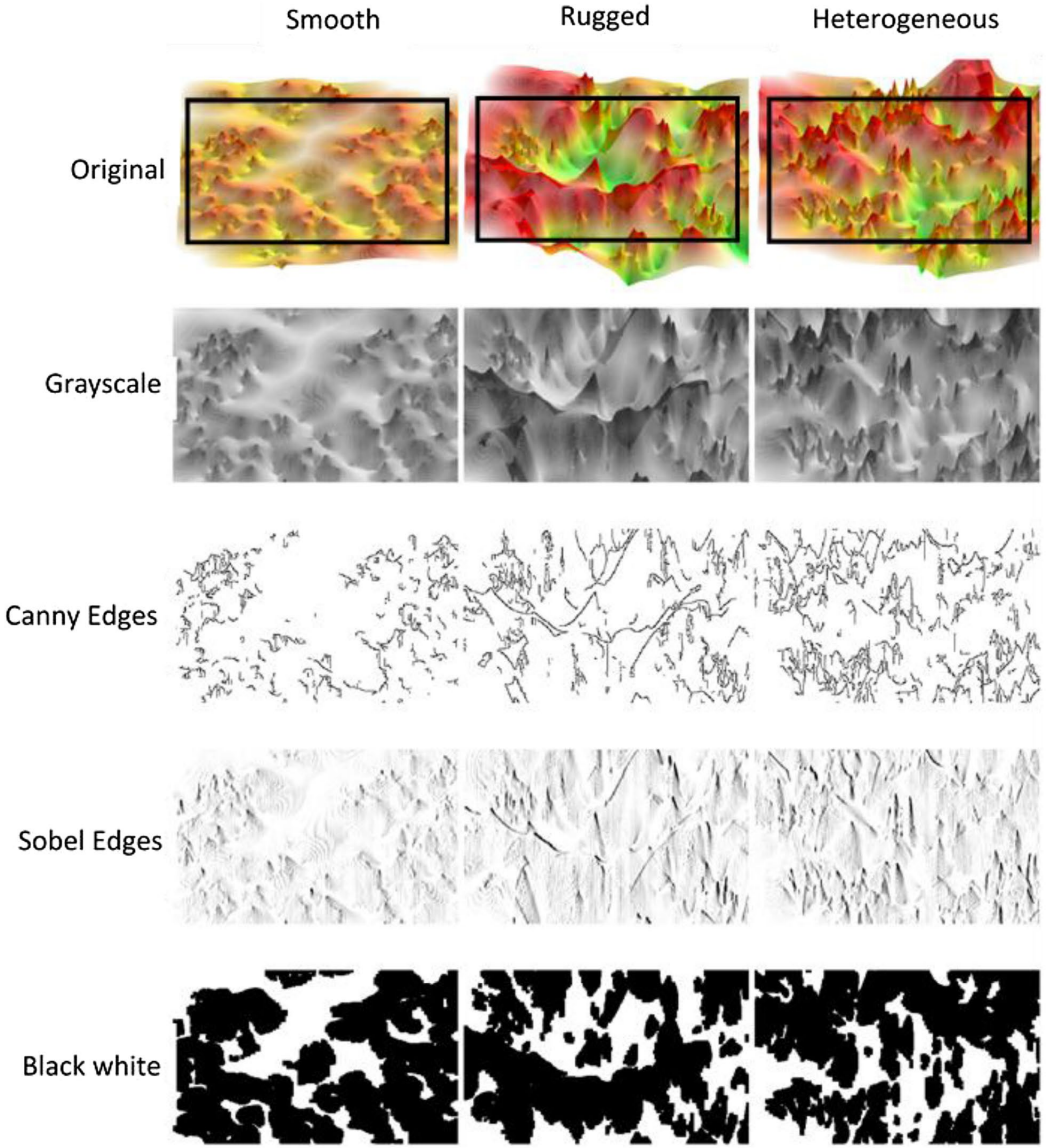
Image variants. From original color-coded 3D ALs, image variants with reduced information content were generated. Shown are examples for activity class ChEMBL2014. Rectangles in the original images delineate cropped images

For each original 3D AL and Ref-AL, images providing different views were generated by systematically varying azimuth (0°, 90°, 180° 270°) and elevation angles (0°, 35°, 65°,90°), as illustrated in Fig. 1. For the elevation angle of 0°, most of the 2D projection information is lost but altitude is accounted for as a topological feature. By contrast, for the elevation angle of 90°, elevation information is only retained through potency coloring. Furthermore, original color images were converted into image variants with reduced information content including grayscale and black and white (b/w) versions as well as images generated from edge detection filters (see below). Exemplary images are shown in Fig. 2.

### Convolutional neural networks

**Fig. 3:**
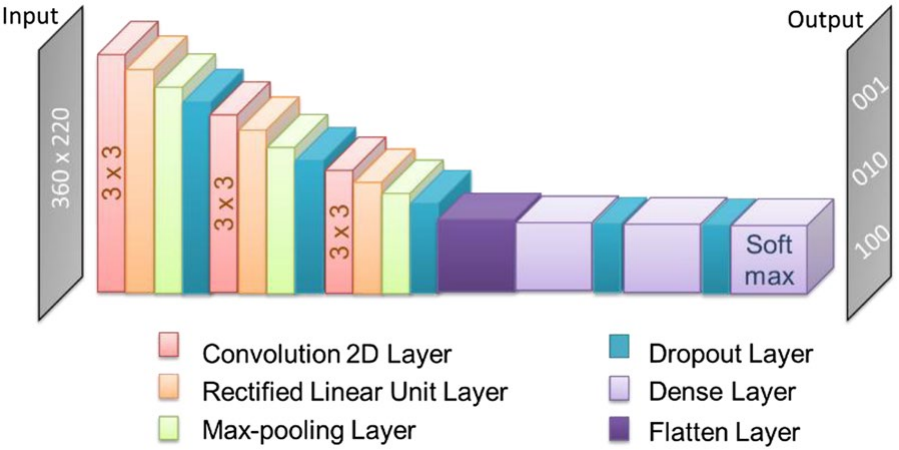
Convolutional neural network architecture. CNN design combining convolution, rectified linear unit, max-pooling, dropout, and dense layers is schematically illustrated

CNNs are deep neural networks characterized by one or more initial convolutional layers. CNNs are popular for image-based analysis tasks [10]. Convolutional layers only connect local neighborhoods of input neurons and perform learnable convolutions on the input data that are identical for each neuron. The output of the convolution layer is passed through a standard rectified linear unit activation (ReLU) layer. This is followed by pooling that combines outputs from local neuron clusters and reduces the dimensions and computational complexity [44]. Multiple convolutional layers can be connected to each other leading to successive reduction of layer sizes. The output of the final convolutional layer is followed by one or more fully connected neuron layers. Dropout layers that randomly deactivate a proportion of neurons are inserted between layers in order to avoid overfitting [45]. A schematic of a CNN is shown in Fig. 3.

### Network architecture

The CNN architecture used herein consisted of convolutional, rectified linear unit (ReLU), max-pooling, dropout, and dense layers, as illustrated in Fig. 3. Three convolutional layers with filter size of 3 × 3 with respect to kernel sizes of 32, 64 and 128 were added to extract image features. Each convolution layer was followed by a rectified linear unit (ReLU), a max-pooling, and a dropout layer. After “flattening” the weights, two intermediate dense layers were added followed by dropout layers. As output, a softmax layer was used to normalize learned weights as a probability distribution. CNN layers were implemented using TensorFlow (version 1.4.1) and Keras (version 2.2.4) [46, 47]. Training data were assembled from 19 randomly selected activity classes. As test sets, all images from the remaining 19 classes were used. CNN hyper-parameters were optimized using internal validation on the basis of an 80% versus 20% split of the training data. Parameter optimization included ReLU alpha over the range 0.0–0.5, dropout rates with values 0.0, 0.1, 0.3, intermediate dense layer sizes of 16, 32, 64, and 128 output neurons, and Adam optimizer learning rates of 100, 10, 1, 0.1, 0.01, 0.001, 0.005, 0.00005, and 0.000005. Each CNN model was trained until convergence was reached, which typically required ~ 20 epochs.

### Alternative machine learning approaches

#### Support Vector Machine

Support vector machine (SVM) is a supervised machine learning algorithm that constructs a hyper-plane *H* in a given feature space to best separate different classes of objects by maximizing the distance (margin of the hyper-plane) between objects having different class labels [24]. The hyper-plane for an $n$-dimensional feature space is defined as:

$$H = \{ x \in {\mathbb{R}}^{n} |w,x + b = 0\}$$

Here, $w \in {\mathbb{R}}^{n}$ w is the weight vector and *b*$\in {\mathbb{R}}^{n}$ is the bias. If linear separation of objects with different class labels is not possible in a given feature space, the data are projected into a higher dimensional space variant where linear separation might become feasible. Therefore, the scalar product $w,x$ is replaced by a non-linear kernel function, thereby circumventing explicit mapping to higher dimensional space. SVM classifiers are trained using a regularization parameter that permits certain misclassification events and penalizes them with a cost factor $C$, which supports model generalization. For multi-class image analysis, multiple one-against-one binary SVM models were trained and the results were combined to yield a final classifier. SVM meta-parameters were optimized using tenfold cross validation including cost factor $C$ with values of 0.01, 0.1, 1 and the kernel (linear, polynomial, or RBF). For SVM training, a total of 79,200 features extracted from images were used.

#### Random forest

RF is a decision tree ensemble classifier that is trained using randomized feature subsets on sub-sampled training data [16]. Herein, RF models were constructed from the subset of 79,200 image features. RF meta-parameters including the number of trees (50 or 100), minimum number of samples (2 or 5), and minimum sample leaf nodes (1 or 3) were optimized using tenfold cross validation.

### Image pre-processing and feature extraction

Original 3D AL images were generated with a resolution of 1200 × 800 pixels. Images were cropped to reduce non-colored areas and outer boundary regions. Cropped images were resized to a resolution of 360 × 220. Grayscale images were obtained as the weighted sum of the red, green and blue channels using weights of 0.299, 0.587, and 0.114, respectively. These calculations were performed using the openCV library version 3 [48–51]. In addition, grayscale images were converted into b/w images by applying binary Otsu’s thresholding [52]. The pixel values of all image matrices were converted into 32-bit floating point format and normalized.

Convolution layers of neural networks can detect feature representations from given image pixel values. However, machine learning approaches such as SVM and RF are not capable of doing so. Therefore, image filters for feature extraction were applied to generate feature sets for SVM and RF calculations.

The Sobel edge operator is a convolution filter for edge detection given by the two convolution matrices:

$$G_{x} = \left( {\begin{array}{*{20}c} { - 1} & 0 & 1 \\ { - 2} & 0 & 2 \\ { - 1} & 0 & 1 \\ \end{array} } \right),\quad G_{y} = \left( {\begin{array}{*{20}c} { - 1} & { - 2} & { - 1} \\ 0 & 0 & 0 \\ 1 & 2 & 1 \\ \end{array} } \right)$$

It introduces an average factor for smoothing random noise of an image and extracts enhanced (thick and bright) edges [53]. Herein, the vertical improved Sobel filter $G_{y}$ of Gao et al. [53] was used. In addition, the Canny edge detector was applied, representing a widely used method for edge detection [54]. The openCV implementation of the Canny edge filter was applied to obtain Canny edges [49]. The resulting row-wise flattened pixel values of edge filters were used as a feature vector. Figure 2 illustrates image variants obtained using the Sobel edge and Canny edge filters. Furthermore, two other filters were used including ORB [55] and Harris boundary features [56] that are less frequently considered for topological features.

### Deriving and evaluating models on image collections

Machine learning models were trained and tested on images viewed from different angles and image variants with different information content generated on the basis of MDS or Neuroscale projections. Images were grouped into different collections, as reported in Table 2. Collections 1–3 included all viewpoints and were distinguished only by the projection method. Collection 1 combined MDS and Neuroscale images while collection 2 and 3 only included MDS and Neuroscale images, respectively. Collections 4–7 focused on different elevation viewpoints combining MDS and Neuroscale projections. As training data, the heterogeneous (original 3D AL), smooth (Ref-AL), and rugged (Ref-AL) variants for all 38 activity classes were used yielding 114 images for each for specific viewpoint and projection. Training was performed on cropped full color, grayscale, and b/w images. Additionally, image variants from Sobel edge or Canny edge filters were used in some settings. For each elevation, four images were generated for azimuth angle of 0°, 90°, 180° and 270°. Depending on the collection, eight to 32 image variants per target were used for model derivation. Training data for all models were extracted features with normalized values obtained from pre-processed images. For training SVM and RF models, pre-processed images were represented as one-dimensional feature vectors without locality information, which was retained in CNNs via the convolutional layers. Training data were assembled from 19 of 38 randomly selected activity classes. As test sets, all images from the remaining 19 activity classes were used.

**Table 2:** Image collections

No.	Projection	Elevation	Azimuth	Number of images
1	MDS, Neuroscale	0°, 35°, 65°, 90°	0°, 90°, 180°, 270°	3648
2	MDS	0°, 35°, 65°, 90°	0°, 90°, 180°, 270°	1824
3	Neuroscale	0°, 35°, 65°, 90°	0°, 90°, 180°, 270°	1824
4	MDS, Neuroscale	90°	0°, 90°, 180°, 270°	912
5	MDS, Neuroscale	65°	0°, 90°, 180°, 270°	912
6	MDS, Neuroscale	35°	0°, 90°, 180°, 270°	912
7	MDS, Neuroscale	0°	0°, 90°, 180°, 270°	912

Different image collections were generated to provide alternative conditions for training and testing of classification models

### Performance evaluation

Classification performance was evaluated based on receiver-operator characteristic (ROC) curves, the area under the ROC curve (AUC), and the confusion matrix. Three standard performance measures were applied including the subset accuracy [57], Matthew’s correlation coefficient (MCC) [58], and the weighted mean F1 score [59]. Subset accuracy is defined as:

$${\text{Accuracy }} = \frac{1}{n}\sum\limits_{i}^{n} {\ {\llbracket Z_{i} = Y_{i} \rrbracket } }$$

where $n$ denotes the number of samples in the test set, $Z_{i}$ is the predicted and $Y_{i}$ is the true label for sample $i$ and $\left[\kern-0.15em\left[ \cdot \right]\kern-0.15em\right]$ is the Iverson bracket taking the value of 1 for a true and 0 for a false predicate [57].

## Results and discussion

### Analysis concept

Our study was designed to investigate image analysis for distinguishing between 3D AL models with different topological features reflecting different SAR characteristics. Graphical SAR analysis has thus far mostly been qualitative and subjective in nature. Therefore, we reasoned that successful classification of 3D AL images according to different topological features via ML would provide a sound foundation for systematically comparing 3D ALs going beyond subjective interpretation of AL models and qualitative analysis of SAR characteristics. We emphasize that AL images do not only provide an attractive representation for SAR visualization, but also a preferred data format for ML-based image classification. AL images are preferred because the underlying AL data matrices are difficult, if not infeasible to use for ML directly. This is the case because the AL data structure consists of an exhaustive pairwise compound similarity matrix and an array of compound potency values that must be combined. For ML, a potency-augmented similarity data matrix would need to be transformed into a fixed-format feature vector or an equivalent representation to enable direct comparison of different AL data matrices for model derivation. This is intrinsically difficult to accomplish for compound data sets of different composition and size for which ALs are usually generated. Challenging data transformations can be circumvented by using standardized images of ALs directly for ML, which also motivated ML image classification from a methodological perspective, in addition to its attractiveness for graphical SAR exploration. Standardizing images inevitably involves investigating different orientations and image views.

In order to assess how different AL features influence the classification performance of ML methods, we did not only study model performance based on different image viewpoints, but also applied two defined image processing strategies. First, for each AL, we generated reference models with increased SAR continuity/smoothness and discontinuity/ruggedness, respectively. This made it possible to determine which topological characteristics were primarily responsible for accurate image classification. Second, for each AL image, variants with successively reduced information content were generated including grayscale, b/w, and edge-filtered image variants, which were also used for training and model building. This made it possible to determine how different image encodings of topological features affect classification performance, in which form distinguishing features were detected by ML models, and which level of image information content was minimally required for classification of images capturing different AL topologies. Using images as direct encodings of ALs for classification and investigating the two image pre-processing strategies via ML represented key components of our proof-of-concept study.

### Activity landscape topology

The top right image in Fig. 2 shows a representative 3D AL. For all 38 activity classes, heterogeneous ALs were obtained that combined smooth and rugged sections in different ways (further examples are provided below). Therefore, to provide topological reference states for assessing the suitability of 3D AL classification, the concept of smooth and rugged Ref-ALs was introduced. For each original 3D AL, Ref-ALs were generated to increase either smooth or rugged AL character through consistently applied potency-based data set modification, as detailed above. For an exemplary 3D AL, the smooth and rugged Ref-AL is shown in Fig. 2 (top). The generation of these 3D AL variants made it possible to formulate well-defined classification tasks to distinguish heterogeneous 3D ALs from smooth and rugged AL reference states and explore features driving machine learning. Feature relevance was further assessed using other AL variants with reduced information content, as also illustrated in Fig. 2.

### Classification of color-coded activity landscape images

First, 3D AL images of 38 activity classes with different combinations of projection and elevation angles and color gradients accounting for compound potency information were investigated. CNN classification models were built for all image collections according to Table 2. SVM and RF modeling were not applicable for this prediction task due to difficulties in algorithmically handling 3D color features. By contrast, CNN models preserved the dimensionality of color gradients. CNN classification performance is summarized in Table 3. CNNs reached a mean accuracy of 0.74 ± 0.1 (mean ± standard deviation) for combined projections and elevations. In addition, MCC values of ~ 0.6 or greater were obtained indicating globally accurate predictions.

**Table 3:** Classification of color-coded images using convolutional neural networks

Collection	CNN	Metric
1	0.74 ± 0.01	Accuracy
	0.74 ± 0.01	F1
	0.61 ± 0.01	MCC
2	0.72 ± 0.02	Accuracy
	0.72 ± 0.02	F1
	0.58 ± 0.03	MCC
3	0.71 ± 0.02	Accuracy
	0.71 ± 0.03	F1
	0.56 ± 0.04	MCC
4	0.73 ± 0.04	Accuracy
	0.73 ± 0.04	F1
	0.60 ± 0.06	MCC
5	0.70 ± 0.04	Accuracy
	0.70 ± 0.04	F1
	0.55 ± 0.06	MCC
6	0.72 ± 0.03	Accuracy
	0.72 ± 0.03	F1
	0.58 ± 0.04	MCC
7	0.75 ± 0.03	Accuracy
	0.75 ± 0.03	F1
	0.62 ± 0.04	MCC

The table reports classification results for CNN models trained and tested on color-coded images. All values reported are averages ± standard deviations over 10 independent trials

**Fig. 4:**
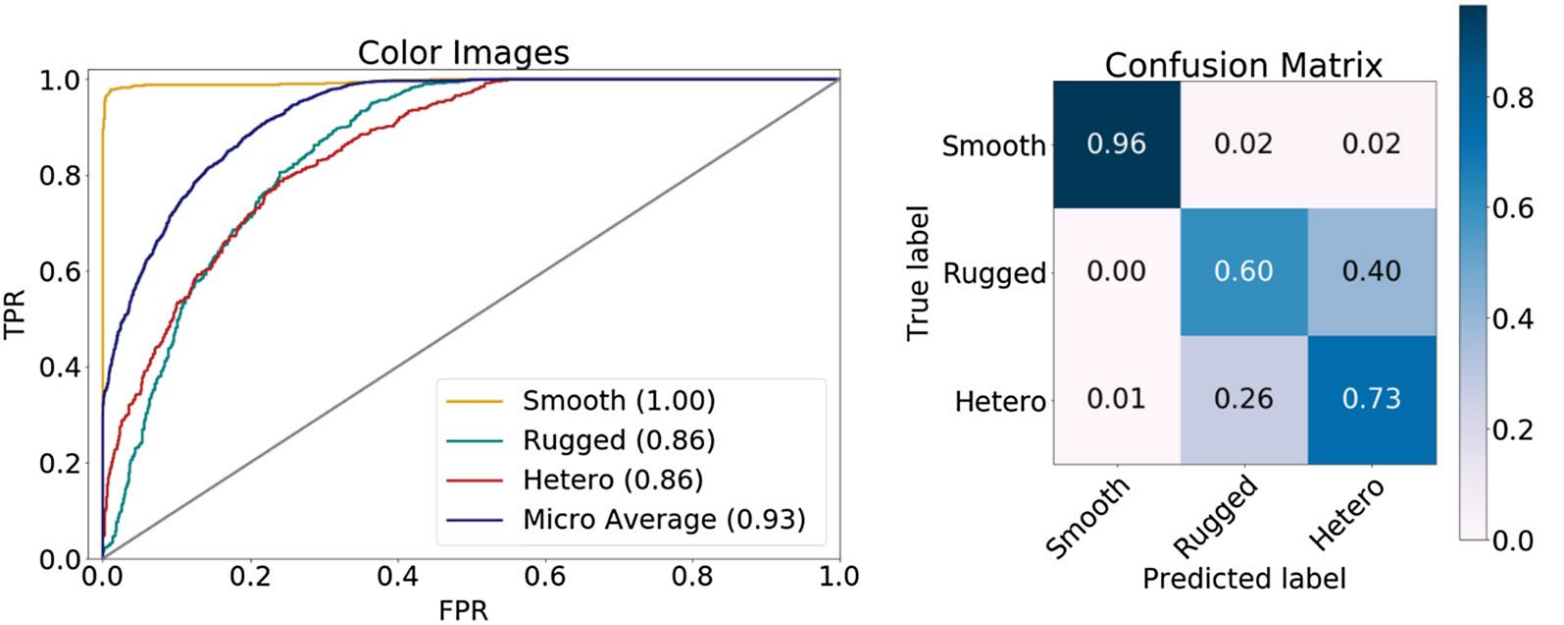
ROC AUC results and confusion matrix for CNN models and image collection 1. On the left, ROC curves for predictions of one versus other classes are shown (yielding a micro average value of 0.93 for all classes). On the right, the confusion matrix is shown for collection 1 color-coded by true positive rates

When classification performance was separately considered for the different image classes, smooth Ref-ALs, rugged Ref-ALs, and heterogeneous 3D ALs from collection 1 achieved ROC AUC values of 1.00, 0.86, and 0.86, respectively, as shown in Fig. 4. In addition, the confusion matrix for all images revealed that CNNs were able to classify images of smooth, rugged and heterogeneous 3D AL variants with a true positive rate of 96%, 60% and 73%, respectively (Fig. 4), reflecting overall accurate predictions.

**Fig. 5:**
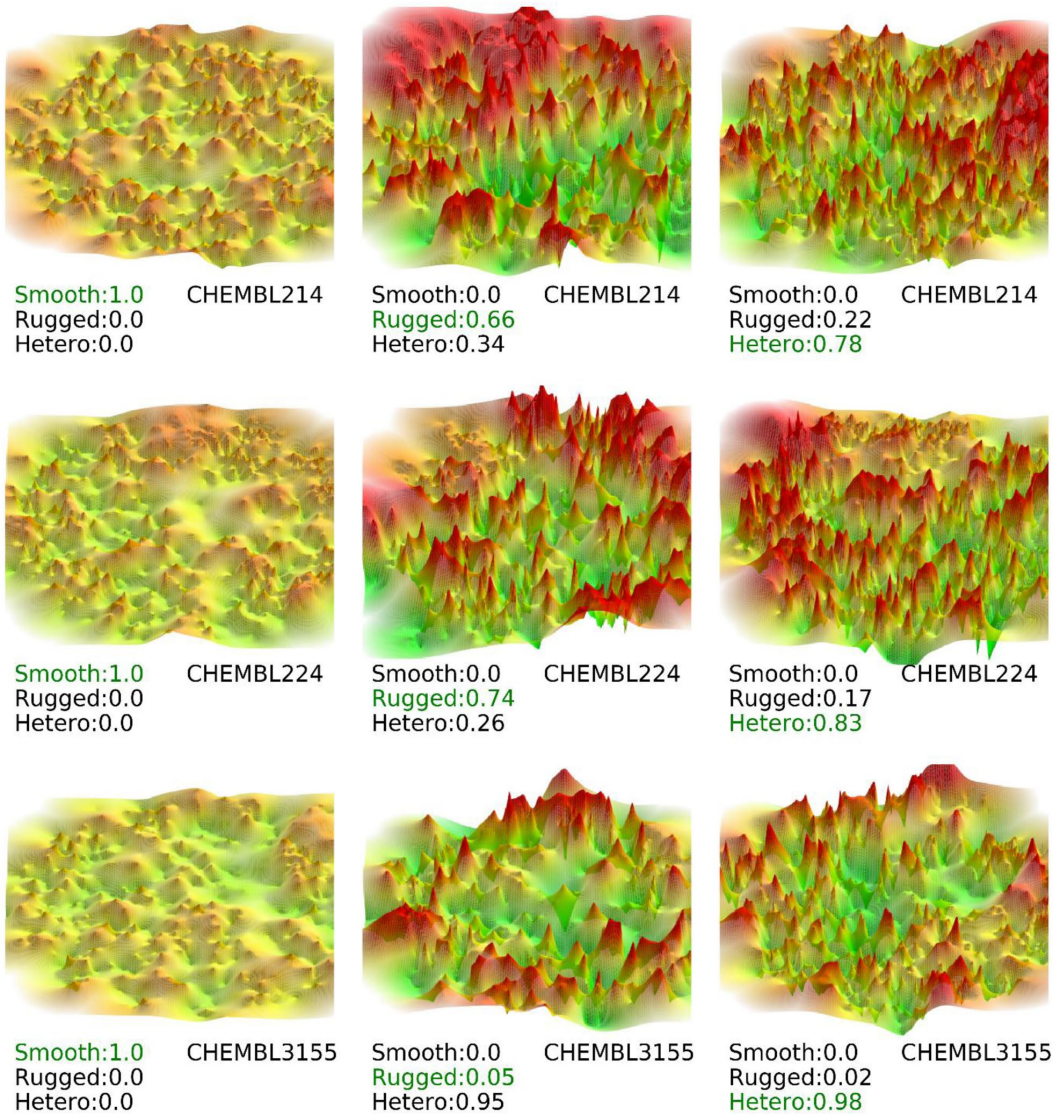
Top CNN prediction probabilities for image collection 1. Results are shown for color-coded images on the basis of Neuroscale projection with azimuth and elevation angle of 90° and 65°, respectively, and three representative activity classes. Correct class labels are shown in green. “Hetero” stands for heterogeneous

Probabilities for class predictions using the best performing CNN model for collection 1 with images taken from the 90° azimuth and 65° elevation angles are shown in Fig. 5.

Predicted class probabilities displayed the general trend that smooth Ref-ALs were consistently predicted with high accuracy, whereas distinguishing between heterogeneous 3D ALs and rugged Ref-ALs often represented a more challenging prediction tasks, resulting in at least slightly reduced accuracy. These observations indicated that the absence of cliffs and associated features in smooth Ref-ALs was a major determinant for correctly distinguishing them from rugged Ref-ALs and heterogeneous 3D ALs.

When comparing different projection methods (collections 1–3), only small differences in performance were observed with only a slight decrease average accuracy of 0.01–0.02 for the individual projections (collection 2 and 3) compared to the collection with combined projections (collection 1). Hence, MDS and Neuroscale projections were readily comparable for classification. CNN models trained separately on different elevations (collection 4–7) performed consistently well. Interestingly, the performance was overall best using 0° elevation angle images (collection 7), with an average accuracy of 0.75 ± 0.03. These projections only visualized altitude profiles of the 3D ALs. These findings also indicated that features associated with cliffs and their absence in smooth Ref-ALs had a major influence on the classifications. By contrast, varying image viewpoints originating from different azimuth and elevation angle combinations were not significantly affecting prediction accuracy, which alleviated the need to establish constant reference frames for 3D AL comparisons.

Collection 4 consisted of top-down views of ALs where potency differences were only distinguished by the color gradient. These views corresponded to 2D heatmap representations of ALs given in four different rotations. In this case, the accuracy of the CNN model was 0.73 ± 0.04 and thus only slightly reduced compared to the profile views of collection 7. This observation was of interest since heatmap views contained the complete information of the AL captured by the color gradient while profile views provided color information and topology. However, in contrast to lower elevation views where some topographical details might be hidden, in heatmaps, no AL features were concealed. Hence, 2D heatmaps and 3D profile views were suitable AL representations for classification of color-coded ALs. This was an encouraging finding in 3D image analysis.

### Models trained on grayscale and black/white image variants

Different from color-coded 3D ALs, it was possible to train SVM and RF models on grayscale and b/w image variants, in addition to CNNs. Classification results for models trained on grayscale image variants are reported in Table 4.

**Table 4:** Classification of models trained on grayscale images

Collection	RF	SVM	CNN	Metric
1	0.57 ± 0.01	0.53 ± 0.01	0.71 ± 0.02	Accuracy
	0.57 ± 0.01	0.54 ± 0.01	0.71 ± 0.02	F1
	0.35 ± 0.01	0.30 ± 0.01	0.56 ± 0.03	MCC
2	0.54 ± 0.01	0.53 ± 0.01	0.70 ± 0.03	Accuracy
	0.55 ± 0.01	0.54 ± 0.01	0.70 ± 0.03	F1
	0.32 ± 0.02	0.29 ± 0.02	0.55 ± 0.04	MCC
3	0.55 ± 0.02	0.53 ± 0.01	0.70 ± 0.03	Accuracy
	0.56 ± 0.01	0.54 ± 0.02	0.70 ± 0.03	F1
	0.33 ± 0.02	0.30 ± 0.02	0.55 ± 0.04	MCC
4	0.54 ± 0.02	0.57 ± 0.03	0.67 ± 0.03	Accuracy
	0.54 ± 0.02	0.58 ± 0.04	0.67 ± 0.03	F1
	0.31 ± 0.03	0.36 ± 0.05	0.51 ± 0.05	MCC
5	0.55 ± 0.03	0.50 ± 0.01	0.68 ± 0.02	Accuracy
	0.56 ± 0.03	0.51 ± 0.02	0.68 ± 0.02	F1
	0.33 ± 0.04	0.25 ± 0.02	0.52 ± 0.03	MCC
6	0.58 ± 0.01	0.53 ± 0.02	0.72 ± 0.03	Accuracy
	0.58 ± 0.02	0.55 ± 0.02	0.72 ± 0.03	F1
	0.37 ± 0.02	0.30 ± 0.03	0.59 ± 0.04	MCC
7	0.69 ± 0.02	0.68 ± 0.01	0.74 ± 0.04	Accuracy
	0.69 ± 0.02	0.68 ± 0.01	0.74 ± 0.04	F1
	0.53 ± 0.03	0.52 ± 0.02	0.62 ± 0.06	MCC

The table summarizes classification performance for color-coded 3D AL and Ref-AL images using RF, SVM, and CNN models trained on grayscale images. All values reported are averages and standard deviations over 10 independent trials

As expected, for CNNs, the loss in color information slightly reduced global classification performance. However, for the combined collection 1, the reduction in accuracy from 0.74 ± 0.01 to 0.71 ± 0.02 was less than one might anticipate. Reduction in performance was largest for high elevation viewpoints (collection 4 and 5) that retained the least altitude information in their projections. Thus, under these conditions, heatmap views from collection 4 were no longer a suitable AL representation, emphasizing the need for applying the color gradient for heatmaps. Moreover, observed differences in model performance between grayscale and color-coded images could be more generally explained. The color gradient used red for low, yellow for intermediate, and green for high potency values while the grayscale was determined as a weighted sum of the red, green and blue channels with weights of 0.299, 0.587, and 0.114, respectively. Thus, yellow resulting from combining red and green appeared brightest, followed by green and red, which yielded darker gray tones representing both high and low high potencies. Hence, dark gray tones did not distinguish between high and low potency values, corresponding to a loss of information. This explained why model performance reduction was largest for the top-down elevation view (0.67 ± 0.03 compared to 0.73 ± 0.04), which exclusively relied on color to differentiate topographical features. By contrast, lower elevation views profited from the presence of topographically detectable peaks and valleys that were retained in the grayscale images, thus confirming relevance of these features for ML.

Furthermore, CNN model performance on collection 1 was superior to RF and SVM models. However, RF and SVM were also able to distinguish between smooth, rugged and heterogeneous 3D AL variants on the basis of grayscale encodings, with a mean prediction accuracy of 0.57 ± 0.01 and 0.53 ± 0.01, respectively. Here, random predictions would correspond to an accuracy of 0.33. CNNs outperformed SVM and RF models for the other collections, with a relative increase in accuracy of 10% or more and consistently higher F1 and MCC values. However, prediction accuracy of all methods improved significantly for the 0° elevation angle images (collection 7) where SVM and RF models reached an accuracy of 0.68 ± 0.03 and 0.69 ± 0.02, respectively, and CNNs of 0.74 ± 0.04. Taken together, the results for models trained on grayscale images revealed that (i) features learned by CNNs from 3D AL images color-coded by potency contributed to the predictions but were not essential and (ii) elevation (peak) information, as emphasized by images from collection 7, was of critical relevance for accurate classifications.

Next, SVM, RF, and CNN models trained on b/w images were investigated. As illustrated in Fig. 2, compared to original 3D AL images, b/w image variants (resulting from binarization of pixel intensities) had drastically reduced information content. Consequently, prediction accuracy of all models trained on b/w image variants was further reduced compared to models trained on grayscale images (Table 5). CNNs retained limited predictive ability for collection 1, with a mean accuracy of 0.62 ± 0.02, but mostly retrained classification performance for images with decreasing elevation angles (65°, 35°, and 0°; collection 5–7). For 0° elevation (collection 7), classification accuracy of SVM and RF models was highest, with 0.68 ± 0.01 and 0.69 ± 0.02, respectively. These observations again emphasized the critical importance of capturing 3D AL altitude information for meaningful image classification.

**Table 5:** Classification of models trained on black and white images

Collection	RF	SVM	CNN	Metric
1	0.48 ± 0.01	0.44 ± 0.01	0.62 ± 0.02	Accuracy
	0.47 ± 0.01	0.45 ± 0.01	0.62 ± 0.02	F1
	0.21 ± 0.02	0.16 ± 0.01	0.43 ± 0.04	MCC
2	0.46 ± 0.01	0.43 ± 0.01	0.61 ± 0.03	Accuracy
	0.46 ± 0.01	0.44 ± 0.01	0.61 ± 0.03	F1
	0.20 ± 0.02	0.15 ± 0.02	0.42 ± 0.04	MCC
3	0.47 ± 0.01	0.46 ± 0.02	0.60 ± 0.02	Accuracy
	0.47 ± 0.01	0.46 ± 0.02	0.60 ± 0.02	F1
	0.20 ± 0.02	0.19 ± 0.03	0.41 ± 0.03	MCC
4	0.45 ± 0.02	0.47 ± 0.03	0.54 ± 0.05	Accuracy
	0.45 ± 0.02	0.48 ± 0.03	0.54 ± 0.04	F1
	0.17 ± 0.03	0.21 ± 0.04	0.32 ± 0.07	MCC
5	0.41 ± 0.03	0.39 ± 0.01	0.70 ± 0.05	Accuracy
	0.41 ± 0.03	0.39 ± 0.01	0.69 ± 0.04	F1
	0.12 ± 0.05	0.09 ± 0.02	0.54 ± 0.07	MCC
6	0.52 ± 0.03	0.51 ± 0.02	0.69 ± 0.07	Accuracy
	0.52 ± 0.04	0.51 ± 0.03	0.69 ± 0.07	F1
	0.29 ± 0.05	0.26 ± 0.03	0.53 ± 0.10	MCC
7	0.69 ± 0.02	0.68 ± 0.01	0.73 ± 0.02	Accuracy
	0.69 ± 0.02	0.68 ± 0.01	0.73 ± 0.02	F1
	0.53 ± 0.03	0.52 ± 0.02	0.59 ± 0.04	MCC

The table summarizes classification performance for color-coded 3D AL and Ref-AL images using RF, SVM, and CNN models trained on b/w images. All values reported are averages and standard deviations over 10 independent trials

### Edge detection in pre-processed images

Unlike CNN models, SVM and RF models cannot directly learn image feature representations from pixel values. Thus, to further evaluate the predictive ability of SVM and RF models to classify 3D AL images on the basis of topological features, Sobel operators and Canny edge filters were applied to all grayscale images. SVM and RF models were then derived using edge-filtered images from half of the activity classes and tested on edge-filtered images of the remaining half of the classes. The classification results for these SVM and RF models are reported in Table 6. For the most part, no further improvements relative to the performance of RF and SVM trained on grayscale or b/w images were observed. In addition, SVM and RF performance did not improve when applying the ORB and Harris boundary feature filters. Overall, the combination of SVM and the Sobel operator was overall preferred but confined in accuracy to 0.60; however, with a notable exception for collection 7. In this case, these SVM models achieved an accuracy of 0.73 ± 0.02 and 0.74 ± 0.01 for the Canny and Sobel filters, respectively. Interestingly, this level of classification accuracy was comparable to the one achieved by CNNs trained on original color-coded 3D AL and Ref-AL images.

**Table 6:** Classification of pre-processed models on the basis of edge detection

Collection	RF		SVM		Metric
	Canny	Sobel	Canny	Sobel	
1	0.48 ± 0.00	0.50 ± 0.01	0.52 ± 0.01	0.57 ± 0.01	Accuracy
	0.48 ± 0.01	0.50 ± 0.01	0.52 ± 0.01	0.58 ± 0.01	F1
	0.23 ± 0.01	0.26 ± 0.01	0.28 ± 0.02	0.36 ± 0.02	MCC
2	0.44 ± 0.01	0.50 ± 0.01	0.50 ± 0.02	0.56 ± 0.01	Accuracy
	0.45 ± 0.01	0.50 ± 0.01	0.50 ± 0.02	0.56 ± 0.01	F1
	0.16 ± 0.02	0.25 ± 0.02	0.25 ± 0.02	0.34 ± 0.02	MCC
3	0.45 ± 0.01	0.49 ± 0.02	0.51 ± 0.02	0.56 ± 0.01	Accuracy
	0.46 ± 0.01	0.49 ± 0.02	0.52 ± 0.02	0.57 ± 0.01	F1
	0.18 ± 0.01	0.24 ± 0.02	0.27 ± 0.03	0.35 ± 0.02	MCC
4	0.43 ± 0.01	0.54 ± 0.03	0.53 ± 0.04	0.60 ± 0.03	Accuracy
	0.44 ± 0.02	0.54 ± 0.03	0.54 ± 0.04	0.60 ± 0.03	F1
	0.16 ± 0.02	0.31 ± 0.05	0.30 ± 0.06	0.40 ± 0.04	MCC
5	0.44 ± 0.02	0.50 ± 0.02	0.47 ± 0.03	0.55 ± 0.02	Accuracy
	0.44 ± 0.02	0.51 ± 0.02	0.49 ± 0.03	0.57 ± 0.02	F1
	0.16 ± 0.03	0.26 ± 0.04	0.21 ± 0.05	0.33 ± 0.03	MCC
6	0.42 ± 0.01	0.50 ± 0.02	0.52 ± 0.03	0.56 ± 0.02	Accuracy
	0.42 ± 0.01	0.51 ± 0.03	0.52 ± 0.02	0.58 ± 0.02	F1
	0.13 ± 0.02	0.25 ± 0.04	0.28 ± 0.04	0.35 ± 0.03	MCC
7	0.61 ± 0.01	0.66 ± 0.03	0.73 ± 0.02	0.74 ± 0.01	Accuracy
	0.63 ± 0.01	0.66 ± 0.03	0.73 ± 0.02	0.74 ± 0.01	F1
	0.42 ± 0.02	0.50 ± 0.04	0.59 ± 0.03	0.61 ± 0.01	MCC

Results are reported for RF and SVM models trained on edge-filtered images and applied to classify such images originating from different activity classes. All values reported are averages and standard deviations over 10 independent trials.

Importantly, edges in pre-processed images resulted from data peaks in rugged regions of 3D ALs. Hence, the classification performance of SVM model on these filtered image variants clearly indicated the critical importance of altitude-dependent topological features for image classification. The sparseness of such features in smooth Ref-ALs rationalized the ability of classification models to distinguish these image variants with very high accuracy from rugged Ref-ALs and heterogeneous 3D ALs. In these two image categories, altitude-dependent topological feature accounting for peaks in 3D ALs were prevalent. Accordingly, rugged and heterogeneous AL variants were more difficult to distinguish from each other. However, even for this classification task, overall accurate predictions were obtained, indicating that machine learning correctly detected differences in relative feature density and feature combinations.

## Conclusions

In this work, we have investigated classification of 3D AL images using machine learning. The study was motivated by the need to complement SAR visualization and graphical SAR analysis with systematic computational assessment of different 3D AL representations. The study concept took into consideration that images also represented a preferred data format for machine learning using 3D AL models of compound data set of diverse composition. Therefore, for 38 different activity classes with significant compound potency variations, we have generated a variety of 3D AL image variants including Ref-ALs designed to emphasize different topological features in a consistent way. These sets of images were classified using ML on the basis of topological features accounting for different SAR characteristics. Original color-coded 3D AL models and corresponding heatmap views were accurately classified using CNN models trained on learned representations, lending credence to the use of such representations. In addition, CNN, SVM, and RF models produced meaningful classification of 3D AL images with models trained on image variants having lower information content. Furthermore, SVM models were able to accurately predict pre-processed images on the basis of edge information representing altitude-dependent features. Thus, investigating a hierarchy of AL representations with successively reduced information content revealed factors that were critical for classification. Taken together, classification of images of different design representing 3D ALs from different viewpoints revealed a pivotal role of elevation-dependent features for accurate image classification, hence providing a diagnostic for the predictions. These features were decisive for distinguishing images of smooth 3D ALs with very high accuracy from images of rugged and heterogeneous 3D ALs. In addition, images of rugged and heterogeneous 3D ALs were also differentiated with meaningful accuracy. Accordingly, on the basis of our proof-of-concept investigation, image analysis is thought to have considerable potential for distinguishing between 3D ALs with different topologies and hence for classifying them on the basis of SAR information they contain. Accordingly, future work will focus on differentiating heterogeneous 3D ALs on the basis of the relative content of SAR continuity versus discontinuity. Classification of such 3D ALs might be attempted on the basis of images capturing differential density of elevation-dependent topological features.

## Data Availability

All calculations were carried out with open source software as specified, except the OpenEye toolkit, for which a free academic license is required. Activity classes, image data, and calculation scripts used herein are freely available from the university cloud via the following link: https://uni-bonn.sciebo.de/s/5XSWARDjTACYvhA.

## Abbreviations

AL: Activity landscape
AC: Activity cliff
AUC: Area under the curve
b/w: Black and white
CNN: Convolutional neural network
Conv: Convolution
GPR: Gaussian process regression
MCC: Matthew’s correlation coefficient
MDS: Multi-dimensional scaling
RBF: Radial basis function
Ref: Reference
ReLU: Rectified linear unit
RF: Random forest
ROC: Receiver operating characteristic
SAR: Structure–activity relationship
SVM: Support vector machine
Tc: Tanimoto coefficient
3D: 3-dimensional
